# From Mechanisms to Application: A Case-Based Review of *Paulownia*-Derived Biochar in Turfgrass Systems

**DOI:** 10.3390/plants15172637

**Published:** 2026-08-28

**Authors:** Marija Koprivica, Marija Simić, Jelena Dimitrijević, Marija Ercegović, Jelena Petrović

**Affiliations:** Institute for Technology of Nuclear and Other Mineral Raw Materials, Franchet d’ Esperey 86, 11000 Belgrade, Serbia; m.petrovic@itnms.ac.rs (M.S.); j.dimitrijevic@itnms.ac.rs (J.D.); m.ercegovic@itnms.ac.rs (M.E.); j.petrovic@itnms.ac.rs (J.P.)

**Keywords:** biochar, *Paulownia* leaf, soil improvement, nutrient storage, water dynamics, soil–plant interactions

## Abstract

Biochar has emerged as a multifunctional soil amendment with the potential to mitigate soil degradation, nutrient loss, and water scarcity. However, its effectiveness depends strongly on feedstock type and production conditions, limiting consistent application across agroecosystems. This review focuses on *Paulownia* leaf-derived biochar (PLB) as a promising feedstock for sustainable soil management. Current knowledge on its production, physicochemical properties, and mechanisms of interaction with the soil environment is synthesized, with emphasis on nutrient storage, water dynamics, and plant responses. To link process-based understanding with practical application, a case-based approach integrates literature evidence with a previously published experimental study evaluating PLB in turfgrass systems under different fertilization and irrigation regimes. The case study illustrates how feedstock-specific properties, including alkaline pH, high cation exchange capacity, mineral enrichment, and a developed pore structure, contribute to enhanced soil functions and turfgrass performance. The combined evidence indicates that PLB may enhance nutrient storage, water availability, and fertilizer-use efficiency, particularly in intensively managed systems. Overall, this review provides an integrated framework for understanding how the physicochemical properties of *Paulownia* leaf-derived biochar are translated into soil functions and agronomic responses, supporting the targeted selection and application of biochar for sustainable soil management.

## 1. Introduction

Soil degradation is a major challenge for sustainable agriculture and ecosystem functioning. Intensive cultivation, excessive fertilizer application, and poor soil management can reduce soil organic matter, accelerate nutrient depletion, and deteriorate soil structure, ultimately limiting soil fertility, water availability, and plant productivity [1,2,3,4,5]. In this context, the concept of soil health has gained increasing importance as a holistic framework for evaluating the capacity of soil to maintain its functions and support plant growth over time [6,7,8]. Soil health is commonly evaluated using a combination of physical, chemical, and biological indicators, including soil structure, moisture, pH, electrical conductivity, nutrient availability, organic carbon, microbial activity, and other properties related to soil functioning [8,9,10]. These indicators are closely interconnected and can be strongly influenced by the application of organic amendments such as biochar [11,12,13]. Therefore, evaluating changes in soil moisture, pH, nutrient availability, and plant physiological responses can provide valuable insights into the effects of biochar on soil functioning and plant performance [6,7,9,10,13]. From this perspective, the effects of biochar should be considered not only in terms of individual soil properties but also within the broader framework of soil health and sustainable soil management [10,11,12]. Among the strategies proposed to maintain and restore soil functions, biochar has gained considerable attention as a sustainable amendment produced by the pyrolysis of renewable biomass. Its carbon-rich composition, porous structure, and reactive surface functional groups contribute to improved interactions with nutrients, water, and soil microorganisms while also promoting long-term carbon sequestration [14,15,16,17,18,19,20,21,22,23,24]. Numerous studies have shown that biochar can raise soil pH and improve nutrient storage, cation exchange capacity (CEC), and water-holding capacity [25,26,27,28]. However, its effectiveness depends on both the feedstock and production conditions, particularly pyrolysis temperature and residence time, which determine its physicochemical properties and agronomic performance [6,7,29,30,31,32,33,34,35]. Consequently, selecting suitable biomass is essential for producing biochars tailored to specific soil applications [30,36]. Since the late 2000s, and particularly during the 2010s, rapidly renewable plant residues have attracted increasing research interest as feedstocks for biochar production [30,36,37].

Among them, *Paulownia* species are particularly promising because of their fast growth, high biomass productivity, and abundant lignocellulosic residues [38,39,40,41,42,43,44,45,46]. *Paulownia* is a fast-growing deciduous hardwood genus with increasing importance in the wood industry, plantation forestry, agroforestry, and biomass production. Its rapid growth and expanding cultivation have generated considerable biomass, including agricultural and forestry residues that can represent an underutilized renewable resource. In recent years, increasing attention has been directed toward the sustainable valorization of *Paulownia* biomass through integrated biorefinery, bioenergy, and other circular-economy approaches [38,39,42,45,46]. Therefore, the conversion of *Paulownia* residues into value-added products such as biochar represents a promising strategy for improving biomass utilization and reducing waste.

Previous studies have reported that *Paulownia*-derived biochars exhibit high carbon content, alkaline properties, well-developed porosity, and abundant functional groups, contributing to improved soil quality and plant growth, especially under water-limited conditions [40,42,44,46]. Despite these promising findings, available studies have primarily focused on reporting individual material characteristics or agronomic outcomes, whereas considerably less attention has been devoted to integrating these observations into understanding how PLB properties govern soil processes and plant responses. Although *Paulownia* leaves (PLs) are generally regarded as low-value biomass, their high cellulose, hemicellulose, and lignin contents favor the formation of stable carbon-rich biochars during pyrolysis [45,46].

Recent reviews have extensively examined the general role of biochar in soil improvement, nutrient cycling, water management, carbon sequestration, and plant growth, emphasizing the importance of feedstock type and pyrolysis conditions in determining biochar functionality [47,48]. Other reviews have addressed the use of specific biomass resources or the broader relationships between biochar physicochemical properties and soil processes. However, these studies generally consider biochar across a wide range of feedstocks and applications and therefore provide limited attention to the specific characteristics and agronomic functionality of *Paulownia*-derived biochar. In particular, an integrated assessment connecting *Paulownia* feedstock characteristics and pyrolysis-derived physicochemical properties with the mechanisms governing nutrient storage, water dynamics, growing medium modification, and plant responses is still lacking. This distinction is important because *Paulownia* biomass, and particularly its leaves, differs from many commonly investigated woody and agricultural feedstocks in terms of mineral composition, ash content, alkalinity, and potential nutrient contribution. Consequently, findings derived from generic biochar reviews cannot necessarily be directly extrapolated to *Paulownia* leaf-derived biochar (PLB). A dedicated synthesis is therefore needed to evaluate how the specific properties of PLB translate into growing medium functions and agronomic outcomes under defined management conditions [49,50].

Accordingly, the aim of this review is not to provide another general overview of biochar effects on soil and plant systems, but to develop a feedstock-specific perspective on *Paulownia*-derived biochar, with particular emphasis on leaf-derived biochar. The review first examines the characteristics of *Paulownia* biomass and the influence of pyrolysis conditions on the resulting biochar properties, and then links these properties to the major mechanisms governing soil pH, nutrient storage, water dynamics, microbial interactions, and plant responses. A further distinctive feature of the present review is the integration of this process-based framework with a previously published experimental study in which PLB was evaluated under contrasting irrigation and fertilization regimes in a turfgrass system. This case-based approach enables the discussion to move beyond a general description of biochar effects and to illustrate how specific physicochemical properties of PLB can be translated into measurable growing medium and plant responses. These responses are particularly relevant to the soil health framework, as they reflect changes in growing medium moisture, chemical conditions, nutrient status, and plant performance, which are closely related to the capacity of a growing medium to sustain plant growth. By integrating the limited *Paulownia*-specific literature with the available experimental evidence and a case study, this review aims to provide a focused framework for evaluating the agronomic potential of PLB and for identifying suitable applications and research priorities.

## 2. Production and Fundamental Properties of Biochar

### 2.1. Formation Process

**Figure 1:**
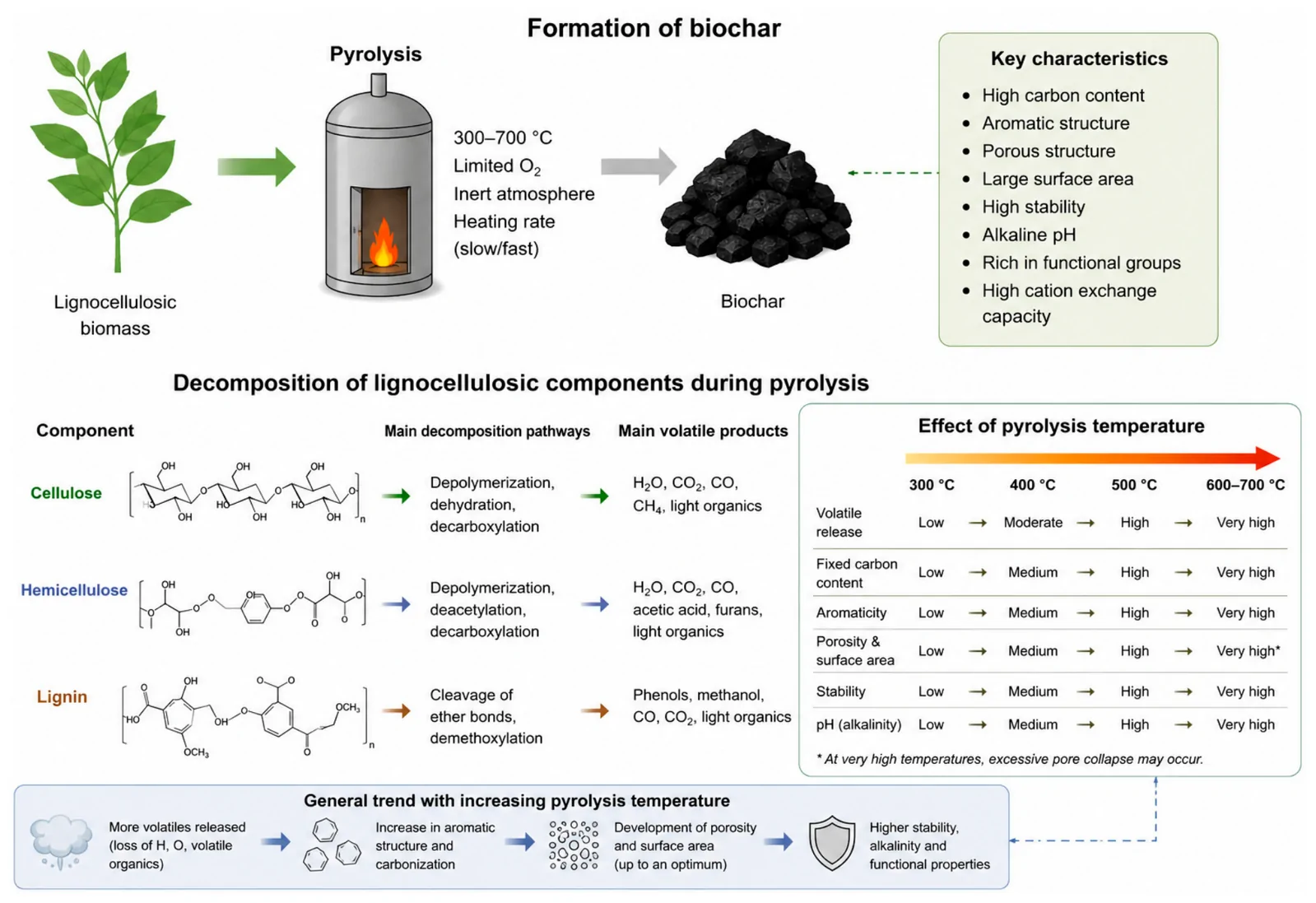
Production and structural evolution of biomass-derived biochar.

Biochar is obtained by heating biomass under oxygen-limited conditions, converting organic matter into a stable, carbon-rich material. Pyrolysis is the most widely used method for its production. Variations in operating conditions and product distribution during the conversion process significantly influence the resulting biochar structure and functionality [51,52,53]. Slow pyrolysis is the most commonly employed method for producing biochar intended for soil applications, due to its higher solid yield and enhanced structural stability [18,19,21,52]. This process involves a sequence of temperature-dependent reactions, including dehydration, depolymerization, and decarboxylation, which collectively transform the original lignocellulosic matrix into a carbon-rich solid product (Figure 1). During thermal treatment, hemicellulose and cellulose decompose at relatively lower temperatures, releasing volatile compounds, while lignin undergoes gradual aromatization, leading to the formation of a condensed and stable carbon structure [54,55].

According to the literature, biochars produced at relatively low pyrolysis temperatures and slow heating rates exhibit properties favorable for soil amendment, including higher ash content, greater CEC, and well-developed porosity while retaining a stable aromatic carbon structure [18,19,56]. During pyrolysis, the release of volatile compounds enriches the fixed carbon fraction and promotes the formation of a porous carbon framework. The resulting physicochemical properties depend on both the biomass composition and process parameters. Key process parameters include pyrolysis temperature, heating rate, and residence time [30,57,58]. Among these factors, pyrolysis temperature has the greatest influence on biochar characteristics. Higher temperatures increase the degree of carbonization, aromaticity, and graphitic ordering, enhancing resistance to microbial degradation and improving long-term stability in soil [58,59,60,61]. However, they also reduce oxygen-containing functional groups, such as carboxyl and hydroxyl groups, which are essential for surface reactivity and ion exchange. Therefore, optimizing pyrolysis conditions requires balancing structural stability and chemical functionality according to the intended application, particularly for soil improvement and nutrient management [47,62]. In addition, differences in the lignocellulosic composition of biomass strongly influence biochar properties, resulting in distinct soil functions among feedstocks.

### 2.2. Common Biomass Feedstocks for Biochar Production and Their Role in Soil Improvement

The agronomic performance of biochar largely depends on the characteristics of the biomass feedstock, as differences in cellulose, hemicellulose, lignin, and mineral composition determine key properties such as carbon stability, porosity, surface functionality, pH, ash content, and nutrient availability [63]. Consequently, feedstock selection is a key factor in designing biochars for specific soil applications. A wide variety of biomass resources have been used for biochar production, including woody biomass, agricultural residues, animal manures, and agro-industrial wastes (Table 1). Woody feedstocks generally produce biochars with high carbon stability and well-developed pore structures, whereas agricultural residues are typically richer in mineral nutrients and functional groups that enhance nutrient storage and soil fertility [9,63]. Biochars derived from animal manures contain high concentrations of ash and plant nutrients, particularly phosphorus (P), potassium (K), calcium (Ca), and magnesium (Mg), making them valuable soil amendments despite the need to consider potential nutrient imbalances and contaminants [64]. Alternative feedstocks, such as sewage sludge and agro-industrial residues, also support waste valorization but require careful quality assessment before agricultural use [9,63,64]. In summary, biochar properties are governed by the combined effects of feedstock composition, pyrolysis conditions, and soil characteristics, resulting in materials with different capacities for improving water retention, nutrient availability, carbon sequestration, and plant stress tolerance. Among emerging feedstocks, *Paulownia* has attracted increasing attention because of its rapid growth, high biomass productivity, and potential to produce biochars with favorable properties for soil improvement. The most commonly investigated feedstocks and their reported effects on soil and plant performance are summarized in Table 1.

### 2.3. Structural, Chemical, and Textural Characteristics

The physicochemical properties of biochar are primarily determined by feedstock composition and pyrolysis conditions. Lignin-rich biomass generally produces biochars with greater aromaticity and structural stability, whereas mineral-rich feedstocks yield biochars with higher ash content, alkalinity, and nutrient availability [76,77,78,79,80]. Pyrolysis temperature is the key factor controlling biochar properties. Higher temperatures promote carbonization and the formation of condensed aromatic structures, resulting in greater resistance to microbial degradation and enhanced long-term carbon storage [81]. However, this increased stability is accompanied by the loss of oxygen-containing functional groups, reducing surface reactivity and CEC. In contrast, lower-temperature biochars retain more hydroxyl, carboxyl, and phenolic groups, improving nutrient storage and interactions with soil particles but decreasing long-term stability [11,82]. Biochar also contains mineral constituents inherited from the parent biomass that influence pH, ash content, and nutrient availability. Most biochars exhibit neutral to alkaline pH due to alkali and alkaline earth carbonates and oxides, enabling them to ameliorate acidic soils [13,83]. They also provide essential plant nutrients, including P, K, Ca, Mg, and, depending on the feedstock, nitrogen (N), which are released gradually and can improve nutrient-use efficiency while reducing leaching losses [81]. The porous structure of biochar, formed through the partial preservation of plant tissues and the release of volatile compounds during pyrolysis, consists of micro-, meso-, and macropores that collectively enhance water retention, nutrient adsorption, soil aeration, and microbial habitats [13,83,84]. Nevertheless, excessively microporous biochars may limit the diffusion of larger molecules, highlighting the importance of both pore size distribution and total surface area in soil applications [81]. Although feedstock type strongly influences biochar properties, direct comparison should also consider pyrolysis conditions, which substantially also affect pH, ash content, and carbon stability. Representative quantitative characteristics of *Paulownia* leaf-derived biochar and selected biochars from commonly investigated biomass feedstocks are therefore summarized in Table 2.

**Figure 2:**
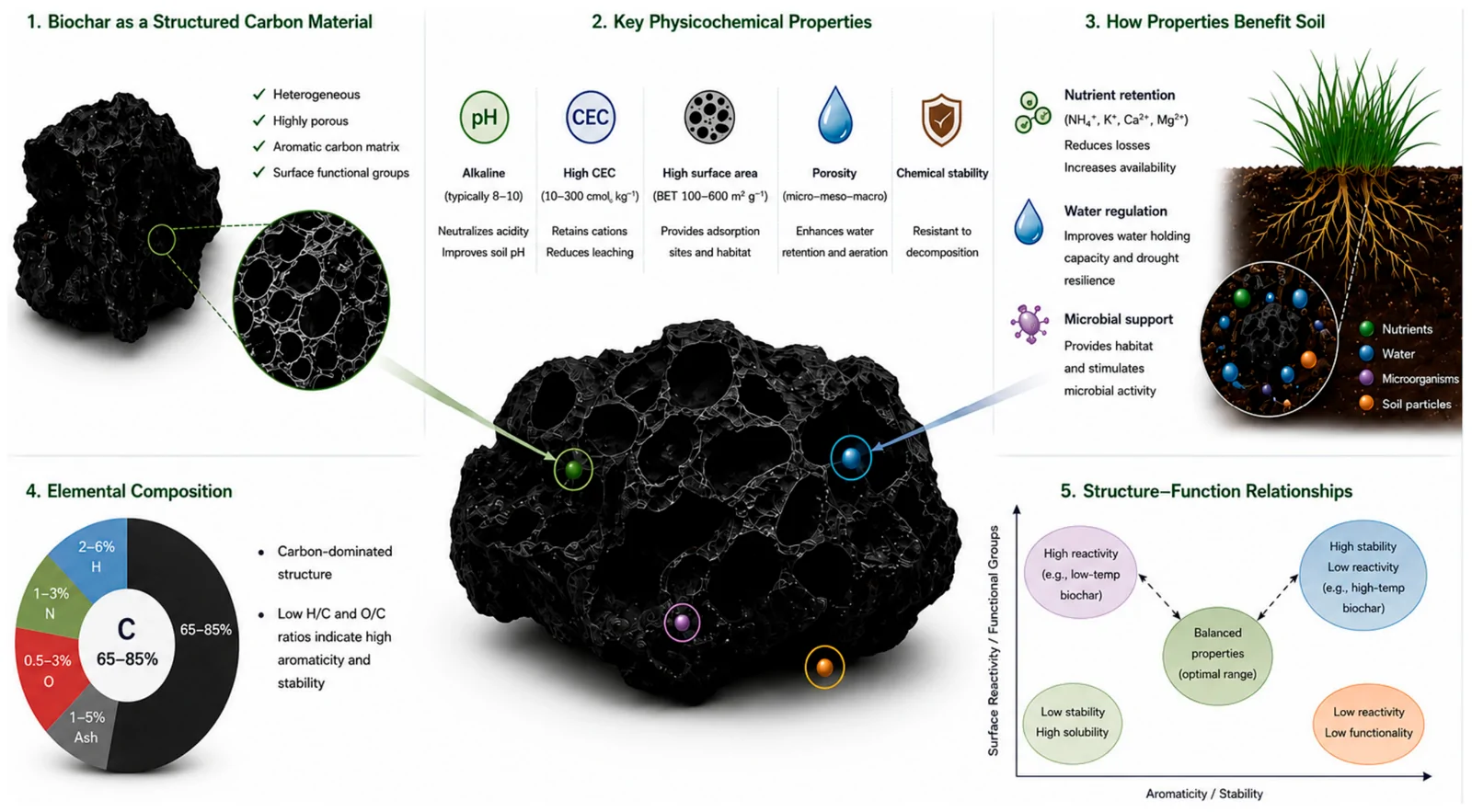
Fundamental properties of biochar relevant to soil improvement.

The quantitative comparison demonstrates that biochar properties are strongly dependent on both feedstock type and pyrolysis temperature. PLB produced at 400 °C exhibited a relatively high CEC and alkaline pH, together with a moderate ash content [44]. These characteristics distinguish PLB from woody biochars, which generally show higher carbon contents and surface areas but lower CEC values, as illustrated by *Paulownia elongata* wood biochar [40]. In comparison, crop- and nutrient-rich feedstocks such as coffee husk can exhibit high CEC and ash contents, whereas lignocellulosic residues such as eucalyptus sawdust and sugarcane bagasse generally produce biochars with lower ash and CEC values under comparable conditions [85]. Therefore, the potential advantage of PLB lies not in universally superior physicochemical properties, but in its particular combination of alkalinity, CEC, and mineral content, which may be advantageous for short-term growing-medium conditioning and nutrient management. Overall, biochar performance in soil applications reflects the interplay among carbon structure, surface chemistry, mineral composition, and pore architecture, which collectively determine its stability, reactivity, and interactions with soil components (Figure 2).

## 3. Mechanisms of Biochar Action in Soil

**Figure 3:**
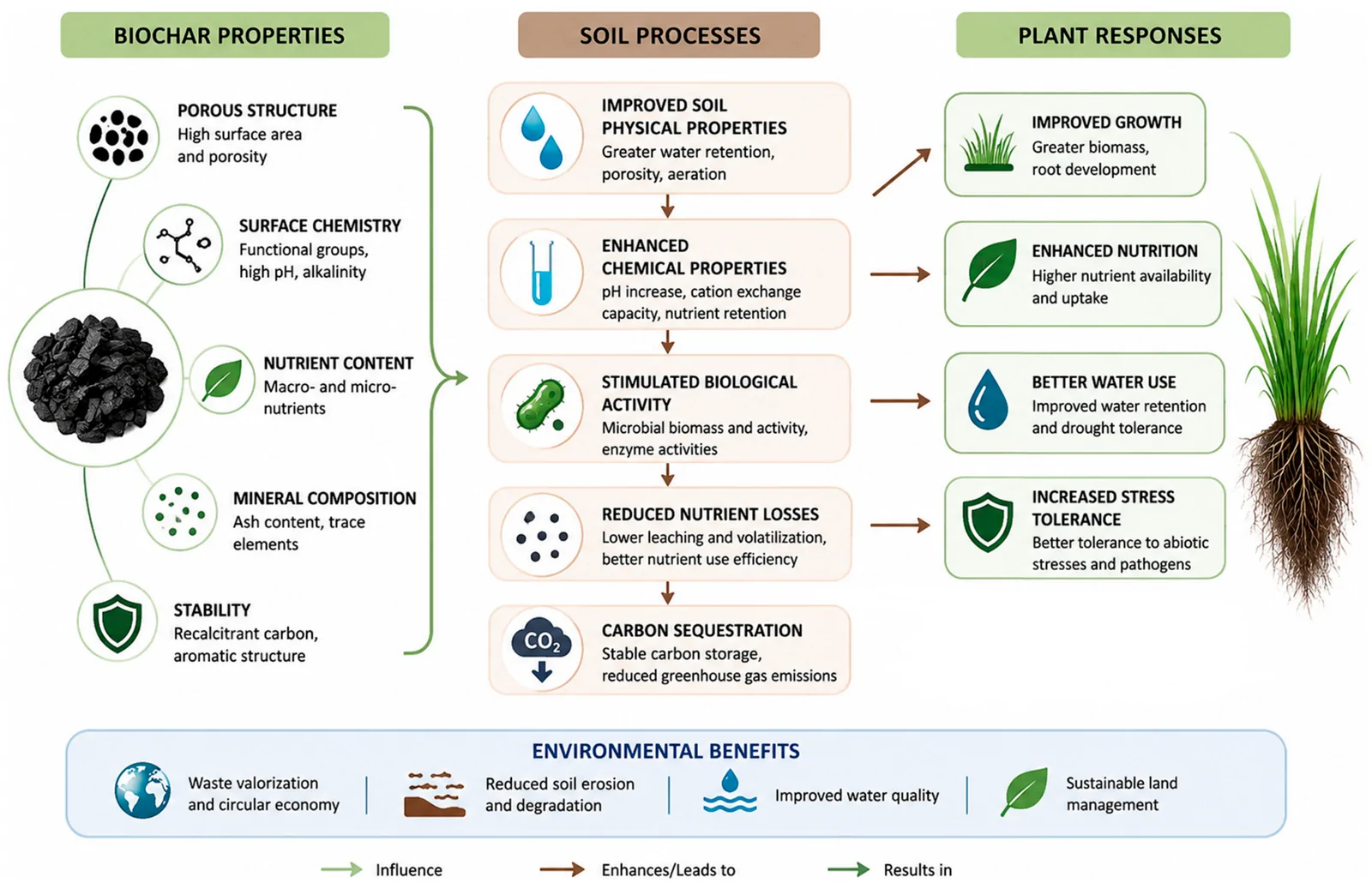
How properties of biochars drive soil processes and improve plant performance.

Biochar influences soil environment through interconnected physical, chemical, and biological mechanisms operating at multiple scales. Its effects arise from the interaction between surface chemistry, pore structure, and material stability, which together regulate nutrient availability, water dynamics, microbial activity, and plant responses (Figure 3) [86]. Understanding the mechanism of these relationships is essential for interpreting its behavior across different soil–plant systems. The following sections summarize the main functional pathways of biochar in soil. Importantly, these mechanisms do not operate independently; changes in pH, surface charge, water availability, nutrient sorption, microbial activity, and redox conditions can interact and collectively determine the magnitude and direction of biochar effects. The following sections summarize the main functional pathways of biochar in soil environment, with particular emphasis on the processes linking biochar physicochemical properties with soil nutrient dynamics and plant performance.

### 3.1. Soil pH Modification

Biochar is widely recognized for its ability to improve soil quality, largely due to its alkaline nature and positive effects on soil fertility and structure. However, excessive increases in soil pH may reduce the availability of nutrients such as P and Mg and negatively affect beneficial microbial activity [13,83]. Therefore, maintaining soil pH near neutral is essential for optimal soil functioning. The wide range of biochar pH (6–10) and CEC (10–100 cmol_c_ kg^−1^) reflects the diversity of biochar materials and highlights the importance of selecting biochars appropriate for specific soil conditions. Biochar modifies soil pH through the release of alkaline mineral components, proton (H^+^) adsorption, and carbonate buffering reactions [87,88]. Its liming effect is mainly attributed to ash constituents, including carbonates and oxides of alkali and alkaline earth metals, which neutralize soil acidity and increase pH [18,87]. Surface functional groups further contribute to pH regulation by interacting with protons, while carbonate buffering helps stabilize soil pH [87,89]. These mechanisms are particularly beneficial in acidic soils, where increasing pH enhances nutrient availability and reduces the mobility of toxic elements such as aluminum and heavy metals [9,87]. In contrast, highly alkaline biochars may undesirably increase pH in neutral or alkaline soils, emphasizing the need to select suitable feedstocks and pyrolysis conditions for specific soil applications [90]. Biochar-induced increases in soil pH may be beneficial in acidic soils, whereas excessive alkalization may reduce the availability of some micronutrients in neutral or alkaline soils [88].

### 3.2. Nutrient Storage and Cycling

Improved nutrient storage is one of the principal mechanisms by which biochars enhance soil fertility. This effect results from the combined action of cation exchange, electrostatic interactions, and physical adsorption within its porous structure. Negatively charged surface functional groups, particularly carboxyl and phenolic moieties, promote the retention of nutrient cations such as NH_4_^+^, K^+^, Ca^2+^, and Mg^2+^, thereby reducing nutrient leaching and improving fertilizer-use efficiency [9,29,32,90]. In addition to electrostatic attraction, nutrient storage may involve specific surface complexation, hydrogen bonding, and interactions with mineral phases present in the biochar ash. Oxygen-containing functional groups, including carboxyl, hydroxyl, and phenolic groups, provide reactive sites whose charge and affinity for dissolved ions depend on soil pH, ionic strength, and the degree of biochar oxidation. As biochar ages in soil, oxidation of its surface can increase the abundance of oxygen-containing groups and consequently modify its CEC and nutrient sorption behavior [82,90]. At the same time, the extensive pore network provides adsorption sites that gradually release nutrients, improving their synchronization with plant demand [13]. The adsorption–desorption equilibrium is particularly important because strong retention does not necessarily imply greater plant availability. Nutrients retained on biochar surfaces may undergo reversible desorption as concentrations in the soil solution decrease, allowing biochar to function as a temporary nutrient reservoir rather than simply an irreversible nutrient sink. Thus, the balance between adsorption strength and desorption controls whether biochar primarily prevents nutrient losses or contributes to sustained nutrient supply [32].

Retention of N is strongly influenced by its chemical form. Ammonium is efficiently retained through cation exchange, whereas nitrate (NO_3_^−^) is less strongly adsorbed and is primarily retained through microbial immobilization or interactions with positively charged surface sites under specific conditions [90]. The weaker sorption of nitrate reflects the predominantly negatively charged surface of many biochars; consequently, nitrate retention is often mediated indirectly through changes in microbial immobilization, soil aggregation, moisture conditions, or localized positively charged mineral and surface sites. Biochar may therefore influence N cycling not only by retaining inorganic N but also by modifying the biological processes responsible for N mineralization, nitrification, and immobilization [31,33]. Together, these mechanisms enhance nutrient cycling while reducing environmental nutrient losses. Biochar also plays an important role in K dynamics. In addition to supplying K directly, it improves K retention in the rhizosphere, increasing its availability for plant uptake [91]. This is particularly important because K regulates enzyme activity, osmoregulation, and stress tolerance, whereas excessive sodium accumulation can disrupt cellular functions [92]. Similar benefits have been reported for Ca and Mg, whose improved availability supports root development, plant growth, and adaptation to environmental stress, especially under nutrient-limited conditions [91]. Phosphorus dynamics are more complex because biochar may either increase or decrease P availability depending on its mineral composition, pH, and surface chemistry. Mineral phases containing Ca, Mg, Fe, and Al can participate in P sorption or precipitation, whereas changes in soil pH and microbial activity may increase P solubilization and plant availability [13,32,47,62,93]. Consequently, the effect of biochar on P should be interpreted in relation to both the biochar mineral fraction and the chemical characteristics of the soil. Overall, nutrient storage by biochar represents a dynamic process governed by the interaction of surface functional groups, mineral phases, pore structure, soil solution chemistry, and microbial activity rather than a single adsorption mechanism [9,32,82].

### 3.3. Water Dynamics in Soil

The incorporation of biochar can improve soil water balance by increasing water-holding capacity, enhancing infiltration, and reducing evaporation losses. These effects are mainly attributed to its porous structure and high specific surface area. Micro- and mesopores retain water via capillary forces, allowing gradual release of water to plants, while biochar incorporation decreases soil bulk density, increases porosity, and promotes water infiltration, thereby reducing surface runoff [94,95]. The effectiveness of this mechanism depends on pore-size distribution: larger pores facilitate water movement and aeration, whereas smaller pores retain water through capillary forces. Therefore, biochars with a balanced distribution of micro-, meso-, and macropores may simultaneously improve water storage and maintain sufficient soil aeration [96]. Biochar may also limit evaporation by modifying soil thermal properties and creating microenvironments that reduce direct water loss. These benefits are particularly important under drought conditions, where improved soil moisture supports plant survival and productivity [95,97]. By maintaining a more stable moisture environment, biochar can also indirectly influence nutrient diffusion and microbial activity, since both processes are strongly dependent on soil water availability [9,33]. At the same time, excessive irrigation can accelerate nutrient losses through leaching and runoff, highlighting the importance of combining biochar application with appropriate water management practices [13,83]. The magnitude of this effect depends strongly on soil texture and biochar pore characteristics, and improvements in water-holding capacity do not necessarily result in measurable increases in plant growth [98].

### 3.4. Interaction with Soil Microorganisms

Biochar influences soil microbial activity by providing suitable habitats for microorganisms and improving soil physicochemical conditions. In addition, biochar contributes to soil organic carbon accumulation by introducing a stable carbon pool, while its small labile carbon fraction can serve as an energy source for microorganisms during the initial stages after application. These processes support microbial activity and soil carbon cycling over time [12,99]. Its porous structure creates protected microhabitats that promote microbial colonization, increase microbial diversity, and reduce exposure to environmental stress [13,83,84]. The pores of biochar can also retain water and dissolved organic compounds, creating microsites with different moisture, nutrient, pH, and oxygen conditions from the surrounding soil matrix [32,33]. Such microscale heterogeneity may promote microbial colonization and facilitate spatially separated processes involved in decomposition and nutrient transformation [33]. Also, biochar enhances nutrient availability, moisture retention, and soil pH, creating favorable conditions for microbial processes such as mineralization, nitrification, and organic matter decomposition. These changes improve nutrient cycling and contribute to long-term soil fertility. Biochar–microorganism interactions may therefore occur through both direct and indirect pathways. Direct effects include microbial attachment to the biochar surface and utilization of labile carbon compounds, whereas indirect effects result from changes in pH, water availability, nutrient concentrations, and the physical protection of organic substrates. These processes can alter the abundance and activity of bacteria and fungi involved in carbon and nutrient cycling, although the direction and magnitude of microbial responses remain strongly dependent on feedstock, pyrolysis conditions, soil properties, and application rate [12,99].

Biochar may also affect soil redox conditions and electron transfer processes, thereby influencing the transformation of nutrients and organic compounds. However, the magnitude of these effects depends on biochar characteristics, soil properties, and environmental conditions [12,29,32,99].

### 3.5. Plant Growth Responses

The improvements in soil physicochemical and biological properties induced by biochar ultimately enhance plant growth and productivity. Reported benefits include greater chlorophyll content, improved root development, enhanced nutrient uptake, and increased tolerance to abiotic stresses such as drought [100,101,102,103]. These responses are largely attributed to improved nutrient availability, greater soil moisture retention, and more efficient N utilization, which collectively promote photosynthesis and biomass production [17,29,100,103]. At the plant–soil interface, these effects are expressed through changes in the rhizosphere, where biochar can modify pH, moisture status, nutrient concentrations, microbial activity, and the distribution of dissolved organic compounds [10,32,33]. By altering these local conditions, biochar may influence root growth, nutrient acquisition, and the activity of microorganisms associated with the root zone. Rhizosphere responses are particularly important because nutrient availability to plants is controlled not only by the total nutrient content of soil but also by the rate at which nutrients are released into the soil solution and transported toward roots [10,32]. Biochar may improve this process by maintaining moisture, retaining mobile nutrients within the root zone, and modifying the chemical environment surrounding roots [32,94,104]. In addition, interactions between biochar surfaces, root exudates, and microorganisms may influence nutrient mobilization and the formation of microsites with enhanced biological activity [10,33]. These effects provide a link between the physicochemical properties of biochar and observed changes in root development, nutrient uptake, chlorophyll content, and plant biomass [101,102].

However, plant responses remain highly dependent on soil type, crop species, biochar properties, and application rate. Excessive doses or unsuitable biochars may produce neutral or even adverse effects, emphasizing the need for site-specific optimization [100,101,102,103]. For this reason, improvements in plant performance should not be attributed solely to direct nutrient supply from biochar; rather, they often reflect the combined effects of nutrient storage, water regulation, pH modification, rhizosphere processes, and microbial interactions [10,32,33].

### 3.6. Impact of Biochar on Fertilizer-Use Efficiency

Mineral fertilizers remain the primary source of N, P, and K for sustaining soil fertility and crop productivity. However, their intensive use is often accompanied by nutrient losses through leaching, volatilization, and surface runoff, reducing fertilizer-use efficiency while contributing to soil degradation, groundwater contamination, and eutrophication. Together with the rising cost of mineral fertilizers, these challenges have increased interest in sustainable strategies that improve nutrient storage and minimize environmental impacts [105]. Biochar has emerged as a promising amendment for enhancing fertilizer efficiency. Its porous structure, large specific surface area, and abundant surface functional groups increase water and nutrient storage, improve CEC, and reduce nutrient losses from the root zone. Consequently, biochar prolongs nutrient availability, enhances plant nutrient uptake, and improves fertilizer-use efficiency, making its integration with conventional fertilization an effective approach for more sustainable soil management [105,106]. This effect can be understood as a shift in the residence time of fertilizer-derived nutrients within the soil–plant system. Nutrients that would otherwise be rapidly lost through leaching or other pathways can be temporarily retained on biochar surfaces or within its pore network and subsequently released through desorption or changes in soil solution chemistry. This creates the potential for a more gradual nutrient supply and greater synchronization between fertilizer availability and plant demand [32,93].

The effectiveness of this mechanism depends on the chemical form of the fertilizer nutrient, biochar surface chemistry, soil texture, moisture regime, and fertilizer application rate. Accordingly, biochar is better considered a nutrient-storage and soil-conditioning amendment than a universal substitute for mineral fertilizers [31,32]. Its greatest potential may occur when biochar and fertilizers are managed together, allowing the biochar to modify the soil environment while mineral fertilizers provide readily available nutrients [78,93]. The principal mechanisms by which biochars improve soil properties, plant growth, and fertilizer-use efficiency are summarized in Table 3.

The mechanisms summarized in Table 3 are interconnected rather than independent. For example, pH modification can alter surface charge and nutrient sorption, water retention can influence microbial activity and nutrient diffusion, and microbial transformations can modify the chemical forms and availability of nutrients stored by biochar [82,99].

### 3.7. Critical Perspective, Knowledge Gaps and Practical Constraints

Despite the well-documented benefits of biochar, its performance under field conditions remains highly variable. Agronomic responses depend on soil type, climate, management practices, and environmental factors such as fertilization, water availability, and temperatures, meaning that benefits observed under controlled conditions are often less pronounced in fertile or neutral soils [107,108,109]. This variability is further influenced by differences in feedstock composition and pyrolysis conditions, which determine key biochar properties, including pH, ash content, porosity, and CEC. In addition, aging in soil can gradually alter biochar surface chemistry and nutrient storage capacity, while short-term effects such as N immobilization or the release of dissolved organic carbon may affect crop responses immediately after application [58,62,81,82,91]. Biochar aging is particularly important for understanding long-term functionality because the material does not remain chemically unchanged after incorporation into soil. Oxidation, dissolution of mineral components, microbial colonization, adsorption of soil-derived organic compounds, and interactions with clay minerals and other soil constituents can progressively modify its surface chemistry and pore accessibility. These processes may increase the abundance of oxygen-containing functional groups and alter CEC and nutrient sorption behavior, while simultaneously changing the accessibility of the underlying carbon structure [82,110]. Long-term carbon stabilization is related to the presence of condensed aromatic structures formed during pyrolysis, which are generally more resistant to microbial decomposition than the original biomass. The degree of carbon persistence depends on feedstock composition and pyrolysis conditions, with greater aromatic condensation and lower H/C ratios generally associated with greater resistance to degradation [58,81,82]. However, carbon persistence should not be interpreted as complete chemical inertness. Over time, a fraction of biochar carbon can be oxidized or mineralized, while the remaining fraction may become physically or chemically stabilized through interactions with soil minerals and aggregation [86]. Thus, the long-term contribution of biochar to soil carbon storage reflects both the intrinsic recalcitrance of its carbon structure and its subsequent transformation and stabilization within the soil environment. The limited number of long-term field studies, particularly those evaluating impacts on soil microbial communities and ecosystem sustainability, remains another important knowledge gap [111,112,113]. Further research is needed to determine how changes in biochar surface chemistry, nutrient sorption, microbial communities, rhizosphere processes, and carbon stability develop over multiple growing seasons rather than only during short-term experiments. Long-term studies should also distinguish between transient responses caused by the initial release of labile compounds and persistent effects associated with the stable biochar fraction [86].

Economic feasibility also varies with local conditions, as production, transport, and processing costs may offset the agronomic benefits [114]. Overall, no single biochar is universally suitable for all soils or cropping systems. Its successful application requires careful selection of feedstock, production conditions, and site-specific management. In this context, characterization of biochar properties should accompany agronomic evaluation, as measurements of pH, CEC, ash content, surface functionality, pore structure, and carbon stability can help elucidate the processes underlying differences in agronomic performance and support more targeted application strategies. Future research should prioritize standardized production protocols, long-term field validation, and evidence-based recommendations tailored to different soil environments.

## 4. Case Study: Paulownia Leaf-Derived Biochar in Turfgrass Systems

Rather than simply restating previously published findings, this case study interprets experimental results through the framework of mechanisms established in Section 3, demonstrating how the physicochemical properties of *Paulownia* leaf-derived biochar (PLB) translate into measurable changes in growing medium properties and turfgrass performance. The discussion is based on our recent study [44], in which PLB was evaluated as a growing medium amendment in a three-month outdoor pot experiment under different irrigation and fertilization regimes. The experiment was conducted using a commercial Floran growing medium (Belgrade, Serbia). Prior to PLB application, the growing medium contained 68.55% moisture, 89.79% organic matter, and 10.21% mineral matter, with total N, P, K, Ca, Mg, Fe, Mn, and Cu contents of 0.52%, 0.023%, 0.046%, 0.16%, 0.15%, 0.29%, 0.0033%, and 0.00052%, respectively [44]. The commercial substrate had an acidic initial pH of approximately 5.0 according to the product specification. These initial characteristics indicate an organic-rich substrate with a defined mineral and nutrient composition, providing a relatively uniform baseline for evaluating the effects of PLB amendment. Therefore, the changes discussed below should be interpreted as responses of the growing-medium–biochar system rather than as effects occurring in an initially uncharacterized soil.

### 4.1. Material Characteristics

The physicochemical characteristics of *Paulownia*-derived biochars depend on the biomass fraction used and the pyrolysis conditions. Previous studies have shown that *Paulownia*-derived biochars can exhibit alkaline properties, developed porous structures, and favorable surface characteristics, supporting their potential for agricultural and environmental applications [40,43,44]. More broadly, leaf-derived biochars are often characterized by relatively high ash and mineral contents, which can contribute to alkalinity, buffering capacity, and nutrient supply, although their properties vary substantially with feedstock composition and pyrolysis temperature [9,63,81]. These characteristics highlight the importance of considering feedstock-specific properties when evaluating the agronomic potential of biochar. In the case study considered here, the *Paulownia* leaf-derived biochar (PLB) contained more than 60% carbon and exhibited an alkaline pH (8.7), relatively high CEC (74.42 ± 0.83 cmol_c_ kg^−1^), and high ash content (17.92%) [44]. The material also showed pronounced buffering and liming properties, as indicated by its acid neutralization capacity (6.38 ± 0.09 mmol g^−1^) and CaCO_3_ equivalent (34.4 ± 0.91%). Pyrolysis concentrated mineral nutrients, particularly Ca, K, Mg, and P, which may contribute to soil buffering and nutrient availability [44]. These characteristics indicate that the investigated PLB was a mineral-rich and chemically reactive biochar, although the magnitude of these properties should not be considered representative of all *Paulownia*-derived biochars because they are influenced by feedstock composition and production conditions [44,81]. The SEM/EDS analysis in the case study showed that PLB retained part of the characteristic cellular architecture of the original leaf tissue while developing a connected porous structure during pyrolysis [44]. Similar preservation of biomass-derived pore structures and the development of accessible surfaces have been reported for other lignocellulosic and leaf-derived biochars, although pore characteristics vary considerably among feedstocks and pyrolysis conditions [9,63,81]. The developed pore network may facilitate interactions with water, nutrients, and microorganisms, while surface functional groups can contribute to nutrient storage and other interfacial processes [9,82]. FTIR analysis further confirmed structural and chemical changes during pyrolysis, including the formation and transformation of functional groups potentially involved in surface interactions [44,82].

Compared with highly lignified woody biochars, leaf-derived biochars may contain a greater proportion of mineral components and may exhibit stronger short-term chemical reactivity, whereas woody biochars can provide greater structural stability and carbon persistence [18,63,81]. Therefore, the characteristics observed for the investigated PLB should be interpreted as a feedstock- and process-dependent example rather than as universal properties of *Paulownia*-derived biochar. Within this context, the combination of alkalinity, mineral content, CEC, porous structure, and surface functionality provides a basis for interpreting the soil and plant responses observed in the case study presented in the following section [9,40,44,63].

### 4.2. Soil and Plant Responses

The application of PLB produced measurable changes in growing medium properties and turfgrass performance during the three-month outdoor pot experiment [44]. Incorporation of PLB increased soil pH, consistent with the alkaline ash fraction and carbonate buffering capacity of mineral-rich biochars. PLB application also affected nutrient availability and electrical conductivity, with the magnitude of these responses depending on the irrigation and fertilization regime [44]. These findings are consistent with broader evidence showing that mineral-rich and leaf-derived biochars can modify growing medium chemical conditions through the release of alkaline mineral components and interactions between nutrients and reactive surface sites [9,81,87]. However, such effects are not universally positive and depend strongly on feedstock composition, pyrolysis conditions, application rate, and initial soil properties [47,62,81]. In particular, the alkalinity and mineral content that may be beneficial in acidic or nutrient-deficient soils can be less advantageous in soils with high pH or fertility, where excessive pH modification or nutrient inputs may alter nutrient availability or soil electrical conductivity [87,88]. The physical properties of PLB also influenced the water status of the growing medium. In the case study, PLB-amended substrates maintained higher moisture contents, particularly under limited irrigation, and this response was associated with more favorable turfgrass performance [44]. Similar effects have been reported for biochars with developed pore structures, although the magnitude of the response varies with pore architecture, particle size, application rate, and soil texture [9,94,97,104]. Biochar-induced improvements in water-holding capacity may therefore be more relevant in coarse-textured or water-limited systems, whereas the effect may be smaller in soils with high inherent water retention. Thus, the response observed in the PLB case study should be interpreted as an example of the broader capacity of porous biochars to modify soil water dynamics rather than as a unique characteristic of *Paulownia*-derived biochar.

**Figure 4:**
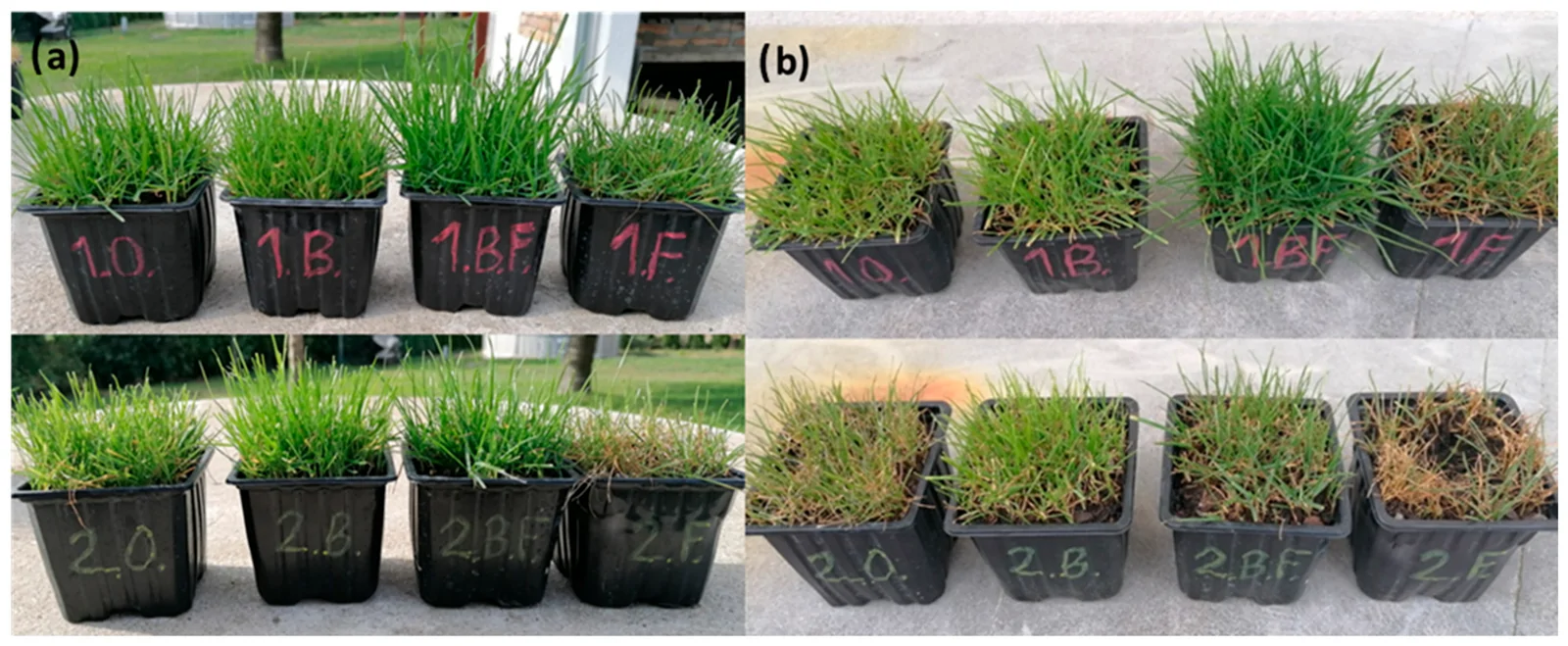
The appearance of the turfgrass after (**a**) one month of planting and (**b**) two months of planting. Representative photographs of turfgrass grown under different treatments two months after planting under (1) regular irrigation and (2) limited irrigation. Treatments: 0—control (no amendment), B—biochar, F—mineral fertilizer, and BF—biochar combined with mineral fertilizer [44].

The observed changes in growing medium conditions were accompanied by improved turfgrass performance, including higher chlorophyll content and improved visual and growth characteristics under selected irrigation and fertilization treatments (Figure 4) [44]. Notably, the response was influenced by the interaction between biochar application, mineral fertilization, and irrigation regime, indicating that the agronomic effect of PLB cannot be considered independently of management conditions. This context dependency is consistent with previous studies showing that biochar effects on plant growth vary with nutrient supply, water availability, application rate, plant species, and the physicochemical properties of both the biochar and the soil [36,47,62,81]. Although positive responses were observed in the PLB case study, biochar application does not necessarily result in measurable improvements in plant performance under all conditions, and neutral or negative responses may occur when changes in pH, salinity, nutrient availability, or water dynamics are unfavorable for the target plant [9,36]. Consequently, PLB may be particularly relevant for intensively managed turfgrass systems where nutrient and water management are closely controlled, but its agronomic performance should be evaluated in relation to the specific soil, plant, and management conditions.

### 4.3. Interpretation of Mechanisms

The responses observed in the PLB case study can be interpreted within the broader process-based framework discussed in Section 3. Rather than acting through a single pathway, PLB may influence the growing medium through the combined effects of chemical regulation, nutrient storage, and physical modification. Its alkaline mineral fraction can contribute to pH regulation and buffering, while its cation-exchange capacity and surface functional groups may facilitate nutrient storage and exchange processes [9,29,82,87,90]. At the same time, the porous carbon matrix can modify water storage and redistribution within the growing medium, which may be particularly relevant under limited irrigation [9,12,104]. Importantly, these mechanisms are not specific to *Paulownia* biochar and have also been reported for other mineral-rich and leaf-derived biochars. Their relative contribution depends on feedstock composition, pyrolysis conditions, biochar dose, soil properties, and management practices [47,62,81]. The stronger turfgrass response observed when PLB was combined with mineral fertilization suggests a potential complementary interaction between biochar-mediated nutrient storage and fertilizer supply, although direct effects on nutrient losses or fertilizer-use efficiency should not be inferred unless these parameters are experimentally measured [44]. Thus, the PLB case study provides an example of how the physicochemical characteristics of a leaf-derived biochar can translate into changes in soil conditions and plant performance. Rather than indicating universal superiority of PLB, the results highlight the importance of matching biochar properties with specific soil, crop, and management requirements [36,47,81]. The main relationships between PLB characteristics, observed responses, proposed mechanisms, and potential applications are summarized in Table 4.

### 4.4. Paulownia-Derived and Related Derived Biochars

Although research on biochar has expanded considerably, studies specifically addressing *Paulownia*-derived biochars remain relatively limited. The available literature nevertheless indicates that the properties and application potential of these materials vary substantially with the biomass fraction and pyrolysis conditions [47,81]. Vaughn et al. [40] reported favorable physicochemical characteristics of *Paulownia elongata* wood biochar for horticultural applications, while Peterson [41] demonstrated its potential as a partial replacement for carbon black in natural rubber composites. More recently, Wang et al. [43] developed hierarchically porous *Paulownia* wood biochar for microplastic removal, illustrating the potential of *Paulownia*-derived carbon materials for environmental remediation. In comparison, the study by Koprivica et al. [44] investigated *Paulownia* leaf-derived biochar (PLB) as a soil amendment and evaluated its effects on soil properties and turfgrass performance under different irrigation and fertilization regimes. Importantly, the limited number of studies specifically focused on *Paulownia* should be considered together with evidence from other leaf-derived biochars. Studies using leaves and other mineral-rich plant residues have generally reported alkaline or near-alkaline biochars, relatively high ash and mineral contents, and substantial variability in nutrient availability, surface properties, and water-related functions [9,18,63,81]. These findings provide a broader context for interpreting the characteristics observed for PLB and indicate that several of its properties, particularly alkalinity, mineral enrichment, and chemical reactivity, are not unique to *Paulownia* but are characteristic of a wider group of leaf-derived biochars. At the same time, leaf-derived biochars do not exhibit uniform behavior. Their physicochemical characteristics are strongly affected by plant species, tissue composition, pyrolysis temperature, residence time, and post-production treatment [9,63,81]. Consequently, the results reported for PLB [44] should be interpreted as a feedstock- and process-specific case study within the broader class of leaf-derived biochars rather than as representative of all *Paulownia*-derived materials. Table 5 summarizes available studies on *Paulownia*-derived biochars together with selected independent studies on related leaf-derived biochars, thereby providing a broader basis for comparison.

### 4.5. Comparison with Other Biochars and Practical Implications

The available evidence indicates that *Paulownia*-derived biochars can exhibit substantially different properties depending on the biomass fraction and production conditions. This variability is consistent with the broader behavior of biochars, for which feedstock composition and pyrolysis conditions strongly influence mineral content, aromaticity, surface functionality, porosity, alkalinity, and nutrient availability [9,63,66,76,79,81]. Accordingly, *Paulownia* biochars should not be considered a uniform material class, and their potential applications should be evaluated according to the properties generated under specific production conditions. Within this context, the PLB evaluated by Koprivica et al. [44] exhibited a combination of alkaline pH, relatively high CEC, and mineral-rich ash, and developed porosity that was associated with favorable changes in the growing medium and turfgrass performance. These characteristics may be particularly relevant to intensively managed systems in which nutrient and water availability are important management constraints. However, the agronomic relevance of these properties depends on soil characteristics, biochar application rate, irrigation regime, fertilizer inputs, and the stability of the resulting biochar–soil interactions. Compared with more structurally stable woody biochars, mineral-rich leaf-derived biochars may provide stronger short-term chemical effects, including pH modification and mineral nutrient release, whereas highly aromatic woody biochars may be advantageous where long-term carbon persistence and structural stability are primary objectives [63,66,76,79,81]. However, these differences represent general trends rather than fixed distinctions, because pyrolysis conditions can substantially modify the properties of both leaf- and wood-derived biochars (Table 6).

From an application perspective, PLB may therefore be particularly relevant to turfgrass and other intensively managed growing systems where rapid modification of soil chemical and water-related properties is desirable. In contrast, biochars selected primarily for long-term carbon stabilization may require different physicochemical characteristics. The most appropriate biochar should consequently be selected according to the intended soil function and management objective rather than according to feedstock category alone. From a practical perspective, however, the transition from experimental application to large-scale use of *Paulownia*-derived biochar requires consideration of economic, logistical, regulatory, and environmental constraints. The feasibility of PLB production will depend not only on the agronomic value of the final product but also on the availability and spatial distribution of *Paulownia* leaf biomass, as well as the costs associated with biomass collection, drying, preprocessing, pyrolysis, storage, and transportation. In particular, the relatively low bulk density and seasonal availability of leaf residues may increase handling and transport requirements compared with more concentrated woody feedstocks. Therefore, decentralized or locally integrated production systems located close to biomass sources may be more feasible than centralized production requiring long-distance transport [115]. Scalability also represents an important consideration. The favorable properties and agronomic responses observed for the PLB case study were obtained under defined laboratory and outdoor pot conditions and cannot be directly extrapolated to industrial-scale production or field application. Large-scale implementation would require consistent feedstock supply, reproducible pyrolysis conditions, standardized product quality, and optimization of energy consumption and process efficiency [63]. In addition, batch-to-batch variability in the mineral composition, ash content, pH, and surface properties of *Paulownia* leaves should be considered when establishing quality specifications for an agricultural PLB product. Standardized characterization and quality-control procedures will therefore be essential for ensuring predictable agronomic performance. Environmental safety and regulatory compliance are equally important for potential commercialization. Before agricultural application, PLB should be evaluated for potentially toxic elements, polycyclic aromatic hydrocarbons (PAHs), soluble salts, phytotoxic compounds, and other contaminants that may be associated with feedstock composition or thermal processing [63]. Although the mineral-rich nature of leaf-derived biochar may provide agronomic benefits, excessive concentrations or application rates could increase soil electrical conductivity, alter nutrient balances, or affect the availability of specific nutrients. Consequently, agronomic benefits should be evaluated together with contaminant thresholds, soil-specific application rates, and applicable quality standards for biochar intended for agricultural use.

Finally, the overall sustainability of PLB should be evaluated over its complete production and application chain. Life-cycle assessment (LCA) studies are needed to determine whether the potential benefits of carbon sequestration, nutrient storage, reduced nutrient losses, and improved water management outweigh the environmental burdens associated with biomass collection, drying, pyrolysis, energy consumption, and transportation [116]. Such assessments should also consider the long-term persistence of biochar carbon and the conditions under which its carbon-storage potential is maintained in soil. Therefore, although *Paulownia*-derived biochar shows promising agronomic characteristics, its large-scale implementation should be supported by integrated techno-economic, environmental, and life-cycle evaluations rather than by agronomic performance alone. These considerations are particularly important for determining whether PLB can provide a scalable and economically viable soil-management strategy under specific regional and agricultural conditions.

### 4.6. Feedstock Characteristics as Drivers of PLB Functionality

Feedstock composition is one of the principal factors governing the physicochemical properties and functional performance of biochar. Woody biomass, which is generally richer in lignin and structural polymers, tends to produce biochars with greater aromaticity and structural persistence, characteristics that may favor long-term carbon stabilization [76,79,81]. However, woody biochars can also exhibit relatively lower concentrations of readily soluble mineral components than biochars produced from mineral-rich plant tissues [9,63].

Leaf-derived biomass represents a distinct feedstock class because leaves commonly contain appreciable concentrations of ash-forming minerals, including Ca, K, Mg, and P. During pyrolysis, these inorganic components become concentrated in the resulting biochar and can contribute to alkalinity, buffering capacity, nutrient release, and interactions with the soil matrix [9,29,81]. Nevertheless, the magnitude of these effects varies among plant species and depends strongly on pyrolysis conditions and the original mineral composition of the biomass.

The PLB investigated by Koprivica et al. [44] illustrates this feedstock effect. Its relatively high ash content, alkaline pH, CEC, mineral content, and developed pore structure were associated with changes in soil properties and turfgrass performance under different irrigation and fertilization regimes. Similar functional characteristics have been reported for other leaf-derived biochars, indicating that the observed behavior of PLB is consistent with broader trends reported for mineral-rich leaf feedstocks rather than being an isolated phenomenon [9,63,81].

Therefore, the relevance of PLB should be considered in terms of the functions required from the biochar rather than as evidence of universal superiority over other feedstocks. Leaf-derived biochars may be advantageous where rapid chemical modification, mineral nutrient supply, and water regulation are desired, whereas woody biochars may be preferable where structural persistence and long-term carbon stabilization are prioritized [63,66,76,79,81]. This feedstock–function relationship provides a useful framework for selecting biochars according to soil properties, crop requirements, and specific management objectives.

### 4.7. Reconciling Positive, Neutral, and Negative Biochar Responses

Although biochar has frequently been associated with improvements in soil quality and plant performance, its effects are not universally positive and may range from beneficial to neutral or, under certain conditions, negative. This variability is a fundamental characteristic of biochar application and reflects the interaction between biochar properties, soil characteristics, environmental conditions, crop species, and management practices. Consequently, the presence of a particular biochar property should not be interpreted as a guarantee of a specific agronomic response.

Biochar alkalinity provides a clear example of this context dependency. Alkaline biochars can effectively increase soil pH and alleviate acidity, particularly in acidic soils. However, the same property may be undesirable in already neutral or alkaline soils, where excessive pH increases can reduce the availability of micronutrients such as Fe, Mn, Zn, and Cu. Similarly, the high ash and mineral content characteristic of some leaf-derived biochars may contribute to nutrient supply and buffering, but excessive application rates or highly soluble mineral fractions may increase soil electrical conductivity and potentially impair plant growth. Therefore, the agronomic value of mineral-rich biochars depends not only on their nutrient content but also on the initial chemical status of the soil and the applied dose [9,63,87].

The effect of biochar on nutrient dynamics is similarly inconsistent among studies. Biochar surfaces can retain nutrients through cation exchange, adsorption, and surface complexation, potentially reducing nutrient losses and increasing nutrient availability. Conversely, strong sorption or interactions with soil minerals may temporarily reduce the availability of some nutrients, particularly when biochar is applied at high rates or when its surface chemistry favors strong nutrient binding [9,29,90]. In addition, nutrient-rich biochars may release substantial amounts of K, Ca, Mg, or P, which can be beneficial in nutrient-deficient soils but may contribute to nutrient imbalance when background soil fertility is already high [32,63].

Water-related effects also depend strongly on the compatibility between biochar pore structure and soil properties. Biochars with high porosity and water-holding capacity may improve water availability in coarse-textured or water-limited soils. However, responses can be much smaller in fine-textured soils with high inherent water retention, and improvements in water-holding capacity do not necessarily translate directly into increased plant growth. Particle size, pore-size distribution, biochar dose, and the degree of contact between biochar and soil therefore influence whether changes in soil water dynamics become agronomically meaningful [9,12,99].

Pyrolysis conditions further contribute to the variability of biochar performance. Increasing pyrolysis temperature generally promotes carbonization, aromaticity, and structural stability, but can simultaneously decrease the abundance of some oxygen-containing functional groups and labile nutrient-containing compounds [63,81,82]. Lower-temperature biochars may retain more surface functionality and readily mineralizable components, but may also be less structurally stable and more susceptible to biological or chemical transformation. Consequently, there is no universally optimal pyrolysis temperature; the appropriate production conditions depend on the intended function of the biochar [9,63,81].

Plant responses are likewise species- and environment-dependent. Positive responses reported for one crop or turfgrass system cannot necessarily be extrapolated to other plant species because plants differ in nutrient requirements, root system, sensitivity to pH and salinity, and capacity to respond to changes in soil water availability. Moreover, biochar effects may become more pronounced under environmental stress, such as limited water availability, while remaining small or statistically insignificant under non-limiting conditions. Interactions with mineral fertilizers can also be positive, neutral, or negative depending on the nutrient status of the soil and the chemical characteristics of the biochar [13,36,64,69].

These inconsistencies do not necessarily indicate contradictory evidence in the literature. Rather, they demonstrate that biochar functions as a context-dependent soil amendment whose effects emerge from interactions among feedstock, production conditions, soil, plant, environment, and management. The PLB case study illustrates this principle: its alkaline, mineral-rich, and porous characteristics were associated with favorable responses under the specific irrigation and fertilization conditions investigated [35], but these effects should not be assumed to occur universally across soils, crops, or application rates. A critical evaluation of biochar performance therefore requires consideration of both positive and non-positive outcomes and emphasizes the importance of matching biochar properties and application strategies to specific soil and management objectives.

## 5. Limitations and Future Perspectives

The present review and case study provide a feedstock-specific perspective on the potential of *Paulownia* leaf-derived biochar (PLB) for soil improvement and turfgrass management; however, several limitations should be acknowledged. First, the experimental evidence underlying the case study is based on a single PLB material produced under one pyrolysis condition and evaluated in a three-month outdoor pot experiment under specific irrigation and fertilization regimes [44]. Although these conditions enabled the relationships between PLB properties and soil and plant responses to be examined under controlled management scenarios, they do not fully represent the complexity and temporal variability of field environments. Consequently, the observed responses should not be considered universally representative of all *Paulownia*-derived biochars, soils, turfgrass systems, or application rates. Long-term field validation is therefore needed to determine whether the beneficial effects observed in the case study persist over multiple growing seasons. Future experiments should evaluate PLB across contrasting soil textures, fertility levels, climatic conditions, and irrigation regimes, while testing a wider range of application rates and fertilization strategies [111,112,113]. Particular attention should be given to changes in biochar properties during aging, including surface oxidation, mineral dissolution, changes in CEC and surface functionality, nutrient release and retention, and interactions with soil organic matter and mineral phases. Monitoring these processes over extended periods would help distinguish short-term responses associated with the initial release of soluble minerals or labile organic compounds from longer-term effects associated with the persistent biochar fraction [58,81,82]. Further research should also assess mechanisms that were not directly quantified in the present case study. Measurements of nutrient leaching, nutrient-use efficiency, microbial community composition and activity, rhizosphere processes, greenhouse gas emissions, and carbon persistence would provide a more comprehensive assessment of the environmental and agronomic consequences of PLB application [86]. In particular, direct measurements of nutrient losses would be necessary to confirm whether the improved turfgrass performance observed under combined PLB and fertilizer treatments is associated with enhanced fertilizer-use efficiency rather than simply with changes in nutrient availability or soil chemical conditions [44,90]. An additional priority is the development of standardized approaches for PLB production and characterization [9,63,81]. Because the physicochemical properties of biochar are strongly influenced by feedstock composition and pyrolysis conditions, differences in *Paulownia* biomass fraction, mineral composition, pyrolysis temperature, residence time, and post-production treatment may result in substantially different materials. Establishing relationships between production parameters, key physicochemical properties, and agronomic responses would facilitate more reliable comparison among studies and support the development of application-specific quality criteria. Emerging approaches may further expand the functionality and practical applicability of PLB in soil and turfgrass systems. In particular, engineered or functionally modified *Paulownia*-derived biochars could be developed to enhance specific properties, such as nutrient storage, water-holding capacity, surface reactivity, or controlled nutrient release, depending on the requirements of the target soil–plant system. The development of biochar-based composites, including combinations with minerals, organic amendments, hydrogels, or other functional materials, may provide additional opportunities to tailor PLB performance and overcome limitations associated with the use of pristine biochar. Another promising direction is the integration of PLB with beneficial microorganisms, such as plant-growth-promoting bacteria and mycorrhizal fungi, to exploit potential synergies between biochar-mediated changes in the rhizosphere and microbial activity. Such microbial-assisted systems could be particularly relevant for turfgrass management, where nutrient-use efficiency, root development, and tolerance to water stress are important considerations. At the same time, precision-agriculture approaches could enable site-specific PLB application by integrating soil properties, turfgrass requirements, irrigation conditions, and spatial variability to optimize application rates and minimize unnecessary material inputs. Data-driven approaches, including machine learning and artificial intelligence, may further support the optimization of biochar production by linking feedstock characteristics and pyrolysis parameters with target physicochemical properties and agronomic performance. These approaches could facilitate the development of application-specific PLB materials rather than relying on a single production condition. From an environmental perspective, future studies should also quantify the long-term carbon sequestration potential of PLB, including carbon persistence, changes during biochar aging, and interactions with native soil organic matter [58,81,82]. Integrating these aspects with water-saving strategies, nutrient-use efficiency, reduced greenhouse gas emissions, and improved resilience to drought and other environmental stresses could position PLB as a component of climate-smart turfgrass and agricultural management. However, the environmental benefits of these emerging approaches should be evaluated through long-term field experiments, life-cycle assessment, and standardized carbon accounting to distinguish potential benefits from those that can be demonstrated under realistic management conditions [116]. Finally, future research should integrate agronomic performance with economic and environmental assessments. As discussed in Section 4.5, the practical feasibility of PLB depends on feedstock availability, production and transportation requirements, process energy demand, product quality, environmental safety, and regulatory requirements. Techno-economic analysis and life-cycle assessment would therefore be valuable for determining whether the environmental benefits associated with carbon stabilization, nutrient storage, and improved water management can be achieved at an economically and environmentally acceptable scale [116]. Such evaluations should consider the complete pathway from biomass collection and preprocessing to pyrolysis, transport, agricultural application, and long-term carbon stabilization.

Overall, future research should move from short-term pot experiments toward multi-season field trials and integrated assessments combining biochar characterization, soil and plant responses, environmental safety, economic feasibility, and life-cycle performance. Such a multidisciplinary approach will be essential for determining the conditions under which *Paulownia* leaf-derived biochar can be translated from a promising experimental amendment into a reliable and scalable soil-management strategy.

## 6. Conclusions

Biochar should be regarded as a multifunctional soil amendment that influences chemical, physical, and biological processes within the soil–plant system. Rather than acting solely as a nutrient source, biochar can modify soil pH, nutrient storage, water availability, and microbial activity. However, its agronomic performance depends strongly on feedstock characteristics, pyrolysis conditions, soil properties, and management practices, indicating that no single biochar is universally suitable for all applications. Within this context, *Paulownia* leaf-derived biochar (PLB) demonstrated considerable potential as a soil amendment due to its alkaline nature, high mineral content, porous structure, and reactive surface properties. The case study showed that PLB improved growing medium chemical and physical conditions, enhanced nutrient storage and fertilizer-use efficiency, and supported turfgrass performance under the tested experimental conditions.

From a practical perspective, the selection and application of PLB should be based on the specific properties and limitations of the target soil, as well as turfgrass requirements, irrigation regime, fertilization strategy, and intended management objectives. Rather than adopting a universal application rate, PLB should therefore be evaluated and optimized according to site-specific soil and environmental conditions. Future research should prioritize multi-season field trials, long-term assessment of biochar aging and carbon persistence, nutrient-use efficiency, microbial and rhizosphere interactions, and environmental safety. Integrating these approaches with techno-economic and life-cycle assessments will be essential for determining the feasibility and sustainability of PLB at larger scales. Overall, *Paulownia*-derived biochar represents a promising feedstock-specific strategy for improving soil and water management in turfgrass systems, while its broader contribution to sustainable agriculture and environmental management will depend on evidence-based material selection, optimized application practices, and long-term validation under realistic field conditions.

## Figures and Tables

**Table 1:** Common biomass feedstocks for biochar production and main characteristics for soil improvement.

Feedstock	Main Characteristics of Resulting Biochar	Potential Agricultural Effect	Ref.
Plant waste	Lower aromaticity; higher H/C ratio; more biodegradable and microbially available.	Increased tomato yield; enhanced carbon mineralization and soil microbial activity; promoted plant growth.	[65,66]
Walnut shell	Highly carbonized; high aromaticity; hydrophobic; highly stable.	Improved soil organic carbon stabilization and carbon sequestration; no significant increase in tomato yield.	[66]
Wood chips	Highly carbonized; aromatic; stable carbon structure.	Enhanced soil organic carbon stabilization; contributed to long-term soil quality improvement; no significant effect on tomato yield.	[66]
Rice husk	High silica content; alkaline pH; porous structure; high CEC.	Improved soil organic carbon, CEC, and available K; reduced bulk density; enhanced soil enzyme activity and beneficial microbial abundance; promoted plant growth and root development.	[67,68]
Wheat straw	Alkaline; porous carbon-rich structure; stable organic carbon.	Increased soil organic carbon, total N, microbial biomass, and nitrate retention; reduced nitrate leaching; improved crop growth and grain yield.	[69]
Maize stover (corn stover)	Carbon-rich; porous structure; stable organic carbon.	Improved soil aggregate stability; increased soil porosity and organic carbon; promoted organo-mineral complex formation; reduced bulk density.	[70]
Coconut shell	High carbon content, porosity, high alkaline pH, high CEC, chemically stable carbon structure	Increased soil pH and CEC, improved nutrient availability and N cycling, enhanced microbial activity and beneficial microbial communities, increased chilli growth and yield, improved soil fertility in acidic soils.	[71,72]
Sugarcane bagasse	High carbon content, alkaline properties, porous structure	Improves soil pH, CEC, and water-holding capacity, supports increased plant biomass and nutrient uptake.	[73]
Fruit-processing residues	High lignin-derived carbon, stable structure, good porosity	Enhances soil carbon content, structure, and water retention. Improves plant stress resistance and root development.	[74]
Animal manure	High ash content, enriched with N, P, K, Ca, and Mg	Provides nutrients, increases microbial activity and soil fertility. Promotes rapid plant growth and nutrient storage.	[64,65,75]
Sewage sludge	High mineral content, nutrient-rich ash fraction	Increases soil organic matter and nutrient supply. Can improve plant productivity when contaminant levels are controlled.	[8]
*Paulownia* leaves	Increased ash content, moderate CEC, alkalinity, liming potential	Improved soil pH, EC and CEC, Enhanced nutrient availability, improved soil fertility and plant growth response.	[44]

**Table 2:** Comparative physicochemical characteristics of biochars derived from *Paulownia* leaves and selected biomass feedstocks.

Feedstock	Pyrolysis T (°C)	pH	CEC (cmol_c_ kg^−1^)	Ash (%)	C (%)	Stability	N, P, K (g kg^−1^)	Ref.
*Paulownia* leaves (PLB)	400	8.7	74.4	17.9	63.4	O/C = 0.11	N: 48.4; P: 5.7; K: 15.7	[44]
*Paulownia elongata* wood	>1000	9.4	27.4	4.1	88.1	O/C = 0.073; H/C = 0.014	-	[40]
Eucalyptus sawdust	350	5.9	10.8	0.9	70.0	-	-	[85]
Eucalyptus sawdust	450	8.0	2.2	0.7	79	-	-	[85]
Eucalyptus sawdust	750	9.3	1.4	1.1	91.0	-	-	[85]
Coffee husk	350	9.7	69.7	13.0	60.5	-	-	[85]
Coffee husk	450	9.8	72.0	13.0	61.3	-	-	[85]
Coffee husk	750	9.8	18.9	20.0	66.0	-	-	[85]
Sugarcane bagasse	350	7.0	4.6	1.9	75.0	-	-	[85]
Sugarcane bagasse	450	8.7	1.8	2.1	82.0	-	-	[85]
Sugarcane bagasse	750	9.7	1.3	2.2	91.0	-	-	[85]
Coconut shell	-	9.7	8.2	-	62.1	-	N: 5.8; P: 0.99; K: 12.7	[72]

**Table 3:** The principal mechanisms by which biochar improves soil properties, plant growth, and fertilizer-use efficiency (the mechanisms and effects discussed in Section 3.1, Section 3.2, Section 3.3, Section 3.4, Section 3.5 and Section 3.6).

Mechanism	Primary Biochar Property	Effect on Soil	Plant Response	Key Influencing Factors
pH modification	Alkalinity, ash	Higher soil pH	Better nutrient availability	Feedstock, soil pH
Nutrient storage	CEC, functional groups	Lower nutrient losses	Higher nutrient uptake	Pyrolysis temperature
Water dynamics	Porosity	Higher water retention	Better drought tolerance	Soil texture
Microbial activity	Pore network	Enhanced nutrient cycling	Improved root environment	Soil microbiome
Fertilizer efficiency	Nutrient adsorption	Reduced nutrient losses	Higher biomass/yield	Fertilizer regime

**Table 4:** Relationship between PLB characteristic, observed responses, proposed mechanisms, and potential application.

PLB Characteristics	Observed Response in PLB Case Study	Proposed Mechanism	Potential Application	Potential Limitation/Context Dependence
Alkaline pH and buffering capacity	Increased soil pH	Alkaline mineral fraction and carbonate buffering	Acidic soils, turfgrass systems	Excessive pH increase in neutral/alkaline soils
High CEC	Changes in nutrient availability	Cation exchange and electrostatic interactions	Fertilized growing media	Strong sorption may reduce availability of some nutrients
Porous structure	Higher moisture content under limited irrigation	Water storage and redistribution	Water-limited turfgrass systems	Effect depends on soil texture and pore characteristics
Surface functional groups	Changes in nutrient availability	Surface adsorption and ion exchange	Nutrient management	Effects depend on biochar chemistry and nutrient species
High mineral content—K, Ca, Mg, P	Increased availability of selected nutrients	Mineral dissolution and buffering	Nutrient-poor or acidic growing media	Potential nutrient imbalance or increased EC at high application rates
Combined biochar + fertilizer application	Improved turfgrass performance under selected treatments	Complementary nutrient storage and fertilizer supply	Intensively managed turfgrass	Response depends on fertilizer rate, soil fertility and irrigation

**Table 5:** Overview of published studies on *Paulownia*-derived biochar.

Feedstock	Pyrolysis Conditions	Main Characteristics	Primary Application	Key Findings	Ref.
*Paulownia elongata* wood	Slow pyrolysis; oxidized and unmodified biochars	Alkaline pH, porous structure, favorable physicochemical properties	Horticultural substrates	Oxidation produced limited improvement; unmodified biochar already suitable for horticultural use	[31]
*Paulownia* wood	Pyrolysis followed by silica milling	Carbon-rich material with good reinforcing properties	Natural rubber composites	Partial replacement of carbon black while maintaining acceptable mechanical performance	[32]
Fast-growing *Paulownia* wood	Controlled pyrolysis	Hierarchically porous structure with high adsorption potential	Microplastic removal	Excellent adsorption performance due to engineered pore architecture	[34]
*Paulownia* leaves	400 °C, 2 h	Alkaline pH, buffering capacity, nutrient-rich ash, improved water retention	Soil amendment and turfgrass cultivation	Improved soil properties and plant performance under different irrigation and fertilization regimes	[35]

**Table 6:** Feedstock-property–function relationships relevant to the selection of biochars for soil and plant applications.

Biochar Type	Key Properties	Advantages	Limitations	Application
Leaf-derived biochar	Higher ash/mineral content, alkaline pH, nutrient-rich surfaces	Nutrient storage, buffering, fast response	Lower long-term stability	Turfgrass, stressed soils
Woody biochar	Higher aromaticity, developed carbon structure, greater structural persistence	Long-term carbon stabilization, structural modification of soil	Lower nutrient availability	Long-term soil conditioning
Agricultural residue biochar	Highly variable mineral and organic compositions	Broad range of nutrient and soil conditioning functions	Strongly dependent on feedstock and production conditions	General soil amendment
Engineered/modified biochar	Enhanced surface area or tailored functionality	Target adsorption of nutrient storage performance	Additional processing, cost and potential environmental implications	Specialized remediation or nutrient-management applications

## Data Availability

The data presented in this study are available upon request from the corresponding author.
